# Comparison of a pharmacovigilance screening heuristic with expert clinical consensus for adverse drug reaction signal classification at a Pakistani tertiary care hospital

**DOI:** 10.12669/pjms.42.7.13236

**Published:** 2026-07

**Authors:** Abdullah Umer, Abdul Basit Afzal, Sher Muhammad Sethi, Ainan Arshad

**Affiliations:** 1 Abdullah Umer, MBBS., Aga Khan University Hospital, Karachi, Pakistan; 2 Abdul Basit Afzal, MBBS., Aga Khan University Hospital, Karachi, Pakistan; 3 Sher Muhammad Sethi, MBBS. Department of Medicine, Aga Khan University Hospital, Karachi, Pakistan; 4 Ainan Arshad, FCPS. MRCP(UK). Department of Medicine, Aga Khan University Hospital, Karachi, Pakistan

**Keywords:** Adverse drug reaction, Drug monitoring, Pharmacovigilance

## Abstract

**Background and Objective:**

Adverse drug reactions (ADRs) have a disproportionately higher burden in low- and middle-income countries where pharmacovigilance infrastructure is under-resourced. Electronic medical records (EMRs) can quantify drug-ADR associations but may require interpretive classification. We compared a statistical heuristic against expert clinical consensus for 82 drug-ADR signals from a Pakistani tertiary care hospital.

**Methodology:**

This is a retrospective analysis used electronic records from the Department of Internal Medicine at Aga Khan University Hospital, Karachi, Pakistan from 2018-2022. In this study logistic regression with false discovery rate correction was applied to EMR data (2018–2022). A four-category heuristic (True Signal, Likely Confounding, Reverse Causation, Null/No Signal) was compared to independent classifications by expert clinicians. Agreement was assessed using Cohen’s κ and Fleiss’ κ.

**Results:**

Heuristic-clinician concordance was 36.6% (κ=0.106, p=0.065). Fleiss’ κ among reviewers was near 0 (p=0.997). Of 52 discordant signals, 69.2% involved experts assigning higher signal-ADR plausibility than the heuristic.

**Conclusion:**

Heuristic and clinical judgment might complement each other. Potential hybrid frameworks warrant prospective evaluation for efficient ADR signal refinement in resource-limited settings.

## INTRODUCTION

Adverse drug reactions (ADRs) are a major cause of morbidity and mortality worldwide. They account for 6-7% of hospital admissions and affect 10-20% of hospitalized patients.^1^,^2^ The ADR burden is disproportionately higher in low- and middle-income countries (LMICs) due to limited pharmacovigilance infrastructure, polypharmacy practices, and minimal resources.^3^ Pakistan, like many LMICs, faces significant challenges in routine systematic ADR monitoring, with reporting being underutilized and often voluntary.^4^ Unlike developed countries with established pharmacovigilance networks, LMICs can often lack the systems in place for comprehensive post-market surveillance of drugs.^5^

Traditional pharmacovigilance heavily relies on spontaneous reporting systems, which suffer from gross underreporting, with higher rates in resource-limited settings^6^, leaving a significant gap for the development and implementation of pharmacovigilance tools that can systematically first-flag potential drug-ADR signals. Additionally, in SRSs, denominators for drug-exposed patients are unknown, underreporting is ubiquitous, and statistical signals do not distinguish causal drug effects from confounding by indication or reverse causation.^7^,^8^ Algorithmically generated signals from SRS, however optimized, therefore ultimately require human expert review to determine their clinical significance and appropriate regulatory action.^9^,^10^

Digitized hospital electronic medical records (EMRs) offer an increasingly important data source for pharmacovigilance. Unlike SRSs, EMRs contain a defined denominator (the population of patients prescribed each drug within the same system), allowing regression-based quantification of drug-ADR associations.^11^,^12^ Previous literature suggests that such data provide relevant contextual information but are most valuable when embedded within structured signal management workflows.^13^ Hence, a persistent challenge in EMR-based pharmacovigilance is that statistically derived associations require interpretive classification before they can inform clinical action.

Some of the more challenging aspects lie at the edge cases, such as confounding by indication, where the clinical indication for a drug prescription may cause drug-outcome associations to reflect the severity or natural history of the treated condition rather than a direct pharmacological effect. Similarly, reverse causation presents a related challenge when high-risk patients both receive particular drugs and experience high rates of specific adverse events, generating inflated odds ratios that reflect the underlying disease process rather than drug toxicity.^14^,^15^

A structured heuristic that systematically applies pre-specified statistical thresholds to categorize associations into categories such as true signals, confounding, reverse causation, and null/no signal categories may offer reproducibility, transparency, and scalability compared to case-by-case expert review. However, no prior study has formally evaluated the classification performance of such a heuristic against a clinical expert consensus derived from independent clinician assessments of the same drug-ADR pairs. The present study addresses this gap. We compare heuristic and expert consensus classifications of 82 drug-ADR associations derived from a Pakistani tertiary care hospital, interpreting the extent, direction, and clinical interpretation of disagreement to inform a more robust signal evaluation framework. We hypothesized that the heuristic would demonstrate modest agreement with expert classification while identifying systematic areas of discordance.

## METHODOLOGY

This retrospective analysis used electronic records from the Department of Internal Medicine at Aga Khan University Hospital, Karachi, Pakistan, from 2018-2022.

### Ethical Approval:

The study was approved by the Institutional Ethics Review Committee (ERC# 2025-9035-35501; Dated: July 15, 2025).

Electronic data was requested and retrieved from the Department of Pharmacy and the Hospital Information Management System. ADR cases were identified using specified ICD-9-CM codes (995.2, E930-E949, E933.1) and ICD-10-CM codes (T36.0X5-T50.9X5, T45.1X5, T50.Z95, T78.2X5, T88.6, T88.7, Y40-Y59). Agents not representing systemic pharmacologic therapies, including topical emollients, nutritional infusions, alternative/herbal preparations and non-drug supportive products, were excluded after independent review by two investigators.

The underlying dataset has been described previously at the therapeutic subclass level in a companion analysis, and is also available upon access request elsewhere.^16^,^17^ The present study extends that work to individual drug-level signal classification with expert validation.

### Operational definitions:

Adverse Drug Reaction (ADR): ADRs were identified using predefined ICD-9-CM and ICD-10-CM codes recorded during hospitalization.

### Drug–ADR Association:

A statistical association between exposure to a specific drug and the occurrence of a coded ADR within the study dataset.

### True Signal:

A drug–ADR association meeting predefined thresholds for odds ratio, ADR frequency, prescription volume, and statistical significance, suggesting a potentially plausible pharmacological relationship.

### Likely Confounding:

Associations with modest effect sizes likely influenced by indication bias, comorbidities, or polypharmacy rather than direct drug toxicity.

### Reverse Causation:

Associations where high ADR rates may reflect underlying disease severity or clinical indication for the drug rather than the drug itself causing the adverse event.

### Null/No Signal:

Associations not meeting predefined heuristic criteria for signal classification.

### Expert Consensus:

The plurality classification assigned by the seven independent clinician reviewers for each drug–ADR pair.

### Signal Concordance:

Agreement between heuristic classification and expert consensus classification.

Drug-ADR associations were quantified using univariate logistic regression giving odds ratios (ORs) with 95% confidence intervals. Benjamini-Hochberg false discovery rate (FDR) correction was applied. Drug utilisation frequency (total prescriptions over the study period) was used as a weighting variable to reduce spurious signals driven by extreme prescribing volumes. From 274 candidate associations, 82 were selected by stratified random sampling across OR strata.

### Drug-level associations were classified using the following thresholds:

- *True signal:* OR > 1.5, ADR rate > 5%, and total uses ≥ 200 (~25th percentile), p-value (FDR) < 0.05
- *Likely confounding:* OR 1.0-1.5, and total uses ≥ 200, p-value (FDR) < 0.1
- *Reverse causation:* OR > 2.0, ADR rate > 25%, total uses < 2000 (~75th percentile), p-value (FDR) < 0.05
- *Null/No signal:* All other associations not meeting the above criteria.

Seven clinicians independently classified the same 82 signals. For each drug-ADR pair, reviewers received the drug name, OR, 95% CI, p-value, and total prescription count. Reviewers were instructed to apply clinical pharmacological judgment. Categories were identical to those used in the heuristic. Responses were anonymous and collected without inter-reviewer discussion. Expert consensus was defined as the plurality classification (most frequent category across all votes). The expert review form is also available as Supplementary Material-1.

Data were stored and managed using Microsoft Excel, and statistical analyses were performed in R (version 4.3.0).^18^ Descriptive statistics were presented as counts and percentages. Heuristic-consensus agreement was assessed by percentage concordance and Cohen’s κ. Inter-rater agreement was assessed using Fleiss’ κ. Disagreement patterns were enumerated by direction.

## RESULTS

The 82 reviewed associations spanned ORs from 0.69-13.43, and prescription volumes from 58-11,498. The heuristic classified 26 signals (31.7%) as True Signal, 22 (26.8%) as Likely Confounding, 18 (22.0%) as Null/No Signal, and 16 (19.5%) as Reverse Causation. Expert consensus classified 55 (67.1%) as True Signal, 14 (17.1%) as Null/No Signal, 11 (13.4%) as Likely Confounding, and only 2 (2.4%) as Reverse Causation. Experts collapsed a majority of heuristic-classified Reverse Causation and Likely Confounding designations into the True Signal category. Heuristic and consensus classifications agreed on 30/82 signals (36.6%). Cohen’s κ was 0.106 (p=0.065), indicating slight, non-significant agreement beyond chance. Fleiss’ κ among the seven individual reviewers was approximately 0 (p=0.997), indicating negligible inter-rater reliability.

Of the 52 (63.4%) discordant signals, disagreements followed systematic directional patterns (Table-I). Most prevalent of these were Heuristic Likely Confounding → Expert True Signal (n=16, 19.5%), concentrated among high-frequency, borderline drugs with ORs of 1.33-1.49 (tramadol, pregabalin, alprazolam, clindamycin, hydralazine). The second most prevalent was Heuristic Reverse Causation → Expert True Signal (n=11, 13.4%), for antimicrobials and immunosuppressants with very high ORs (ethambutol OR=8.41, carbimazole OR=6.70, isoniazid OR=6.30, amphotericin B OR=5.89, tacrolimus OR=5.76). A third pattern, Heuristic Null/No Signal → Expert True Signal (n=9, 11.0%), occurred where sub-threshold ORs (range 0.69-1.72) were deemed pharmacologically credible despite failing statistical thresholds.

**Table-I:** Patterns of disagreement between the statistical heuristic and expert consensus classifications (n=52 discordant signals out of 82 reviewed).

Disagreement Type (Heuristic → Expert)	n (%)	Representative Examples (OR)	Interpretation
Likely Confounding → True Signal	16 (19.5%)	Tramadol (1.49), Pregabalin (1.48), Alprazolam (1.46), Clindamycin (1.37), Hydralazine (1.45)	Heuristic attributes moderate ORs to indication bias.
Reverse Causation → True Signal	11 (13.4%)	Ethambutol (8.41), Carbimazole (6.70), Isoniazid (6.30), Amphotericin B (5.89), Tacrolimus (5.76)	Experts identify well-characterised drug-class toxicities possibly misclassified by the Heuristic.
Null/No Signal → True Signal	9 (11.0%)	Hydroxyzine (1.72, 1.34, 1.02), Enoxaparin (0.94), Tizanidine (1.11)	Sub-threshold ORs dismissed statistically.
True Signal → Null/No Signal	6 (7.3%)	Epinephrine (3.89), Phytomenadione (2.61), Pheniramine (2.61), Gelatin (2.56)	Statistically significant associations deemed implausible.
Reverse Causation → Likely Confounding	4 (4.9%)	Filgrastim (13.43), Acetaminophen IV (8.81), Magnesium Chloride (6.72), Tigecycline 50mg (6.24)	Possible difficulty distinguishing mechanisms in critically ill patients.
Other patterns	6 (7.3%)	Ranitidine, Atorvastatin, Drotaverine, Seretide, Menaphthone, Ramipril	Minor bidirectional discordance across LC/Null/RC boundaries; all involve borderline ORs or atypical prescribing contexts

OR values in parentheses are from the primary logistic regression model. LC, Likely Confounding; RC, Reverse Causation.

The converse pattern, Heuristic True Signal → Expert Null/No Signal (n=6, 7.3%), involved agents such as epinephrine (OR=3.89) and phytomenadione (OR=2.61). Four signals (4.9%) showed disagreement between Reverse Causation and Likely Confounding (filgrastim, acetaminophen IV, magnesium chloride, tigecycline). Collectively, 36/52 discordant pairs (69.2%) involved the expert panel assigning a higher-plausibility category than the heuristic, indicating that the heuristic was systematically more conservative. The complete list of evaluated signals, along with heuristic and expert classifications, as well as disagreements, is available as Supplementary Material-2.

## DISCUSSION

This study provides the first formal evaluation of agreement between a structured statistical heuristic and an expert clinical consensus in the classification of drug-ADR signals derived from hospital EMR data. The low overall concordance (36.6%, κ=0.106) and near-zero inter-rater reliability among clinicians (Fleiss’ κ approximately 0) together reveal that neither approach, in isolation, is sufficient for systematic pharmacovigilance in resource-constrained settings.

When the heuristic classified signals as ‘Likely Confounding’ and experts overrode this to ‘True Signal’, these discordant cases were concentrated among high-frequency drugs (tramadol, pregabalin, alprazolam) whose ORs fell between 1.33 and 1.49. These are agents with well-characterized adverse effect profiles.^19^ Experts appear to have applied this background knowledge to override the heuristic’s designation of confounding by indication, likely correctly identifying that these (moderate OR) signals represent established causal drug effects.

Additionally, agents such as isoniazid, ethambutol, amphotericin B, carbimazole, and tacrolimus all have very high ORs (5.76-8.41) alongside very high ADR rates, exactly the pattern the heuristic was designed to attribute to drug toxicity. Isoniazid-induced hepatotoxicity and peripheral neuropathy, ethambutol-induced optic neuritis, amphotericin B-induced nephrotoxicity and electrolyte disturbances, carbimazole-induced agranulocytosis, and tacrolimus-induced nephrotoxicity and neurotoxicity are all recognized, mechanism-based adverse effects.^20^-^23^ These two main disagreement patterns both point to the fact that while the heuristic works well with clear-cut signals, its performance with edge cases might not be optimal, and thus, could most benefit from clinician review.

Epinephrine and phytomenadione carry high ORs in this dataset, but both are prototypical rescue or reversal agents administered preferentially in the context of existing severe reactions or clinical deterioration.^24^,^25^ It should be noted, however, that in this scenario, the most accurate classification likely would have been Reverse Causation, which the clinicians did not attribute to these signals.

The near-zero Fleiss’ κ among the seven clinicians suggests low inter-rater agreement in categorical assignments, highlighting the inherent subjectivity and complexity of ADR signal interpretation. Thaker et al. evaluated agreement between three widely used causality assessment methods (WHO-UMC, Naranjo, and Liverpool algorithms) and found moderate to poor κ values ranging from 0.14 to 0.86, with systematic variation in both sensitivity and specificity between methods and raters.^26^ The signal-level classification task in the present study, where reviewers were asked to assign probabilistic causal categories rather than apply a structured algorithm, places even greater demands on subjective pharmacological reasoning, which may additionally explain the lower agreement.

The complementarity of the heuristic and clinical judgment, as revealed by the directional pattern of disagreements, suggests a natural division of labor that could form the basis of a hybrid pharmacovigilance workflow. The heuristic excels at rapid, reproducible, and scalable classification of a large signal pool into rough categories. It performs well where statistical patterns are unambiguous (clear null associations, extremely high-frequency well-powered exposures, or associations that cleanly satisfy predefined thresholds). Critically, it provides a structured and auditable classification that reduces arbitrary inter-rater variation for signals that do not require high pharmacological reasoning.

Expert review, therefore, adds irreplaceable value when pharmacological prior knowledge is required to distinguish a true mechanism-based toxicity from statistical noise or non-causal confounding. A hybrid framework in which the heuristic first stratifies a signal pool into tiers, and expert review is then sought, could meaningfully reduce reviewer burden while preserving the pharmacological sensitivity that the heuristic lacks. The framework proposed here, heuristic triage followed by targeted expert review, is particularly well-suited to resource-limited settings because it reduces the per-signal review burden on a small expert workforce. Rather than requiring seven clinicians to independently classify hundreds of associations, a validated heuristic could reduce the field of signals requiring expert attention to a focused subset of ambiguous or high-priority cases. This has operational implications for how pharmacovigilance functions might be embedded within clinical quality improvement teams at hospitals in LMICs that are building capacity.

### Limitations

Our study has important limitations. The heuristic was designed and applied to the same dataset from which the signals were derived, and as such, prospective validation on an independent dataset is needed. The expert panel, while consisting of seven practicing clinicians, was constituted at the same institution, and may not represent the full range of clinical pharmacological expertise. The information provided to reviewers also limits the ecological validity of the expert classification. The use of univariate logistic regression without covariate adjustment is a known limitation of the primary signal detection methodology.

## CONCLUSION

Clinician-only classification is insufficiently reproducible for systematic pharmacovigilance. Our findings support the conclusion that the heuristic and clinical judgment could be complementary in which the heuristic stratifies signals for targeted expert review. This, however, warrants prospective evaluation as a more efficient, reproducible, and scalable approach to ADR signal refinement in resource-limited settings such as Pakistan.

### Recommendations

Future work should prospectively evaluate a hybrid heuristic-expert framework, explore the addition of clinical covariates and comorbidity data to the expert information package, and assess improved ways for pre-classification to the burden on expert reviewers without sacrificing sensitivity for true pharmacological signals.
